# Thermodynamic and Electrochemical Characterization of Nd* (III) Ion Diffusion in (LiF-CaF_2_)-Nd_2_O_3_ Molten Salts

**DOI:** 10.3390/ma18030706

**Published:** 2025-02-06

**Authors:** Kailei Sun, Linsheng Luo, Xu Wang

**Affiliations:** School of Metallurgical Engineering, Jiangxi University of Science and Technology, Ganzhou 341000, China; skl0323@163.com (K.S.); luo735373347@163.com (L.L.)

**Keywords:** molten salt, rare earth metal, ion mobility number, radial distribution function, diffusion coefficient

## Abstract

Data on the diffusion and migration characteristics of rare earth metal ions in fluoride molten salt systems are crucial for optimizing the electrolytic preparation of rare earth metals and alloys. This study investigated the solubility, conductivity, and density of the (LiF-CaF_2_)_eut._ system saturated with Nd₂O₃ using the isothermal saturation method, conductivity cell constant variation, and the Archimedes method, respectively. Employing the Hittorf method’s principles, a three-compartment electrolyzer was designed to determine the mobility number of dissolved Nd* (III) ions in the saturated (LiF-CaF_2_)_eut._-Nd_2_O_3_ system. The radial distribution function was computed via ab initio molecular dynamics, and the self-diffusion coefficient of ions in the system was analyzed. Utilizing the Nernst–Einstein equation, the diffusion coefficient of Nd* (III) ions was calculated. The solubility, conductivity, and density of the saturated (LiF-CaF_2_)_eut._-Nd_2_O_3_ system exhibit linear variation within 1173–1473 K. The mobility number of solvated Nd* (III) ions increases linearly with temperature, displaying nonlinear variation with potential within 3.5–4.5 V, and gradually decreases after reaching a maximum of 4.0–4.25 V. The radial distribution function reveals the highest diffusion and mobility barriers for Nd* (III) ions, with solvated O* (II) ions presenting the most significant hindrance. The Nd* (III) ion diffusion coefficients linearly increase with temperature (1123–1373 K) under specific potential conditions (3.5–4.5 V) but exhibit nonlinear changes with potential (3.5–4.5 V) under fixed temperature conditions (1123–1373 K), then decrease after peaking within 4.0–4.5 V. The diffusion coefficients of Nd* (III) ions are sensitive to potential changes.

## 1. Introduction

The demand for rare earth metals and alloys is increasing annually, driven by the expansion of their application fields. Molten salt electrolysis, a primary method for preparing rare earth metals, requires optimization to align with the “dual-carbon” strategy, which refers to the dual goals of reaching peak carbon emissions by 2030 and carbon neutrality by 2060 [1]. The fluoride–rare earth oxide system, serving as the molten salt carrier for electrolysis, necessitates a clear understanding of its physicochemical property changes to enhance electrolysis efficiency. The transport and diffusion properties of rare earth metal ions in their dissolved state are crucial for optimizing the electrode reduction process. The diffusion coefficient is a vital parameter for determining the transport properties of active rare earth ions and directly affects the conductivity, reaction rate and mass transfer efficiency of the molten salt system. The investigation of this parameter holds significant theoretical and practical value for elucidating the migration and diffusion behaviors of rare earth ions [2,3].

The current research methods for addressing the transport-diffusion properties of active ions in molten salt systems cover a variety of techniques such as experiments, theoretical calculations and simulations [4]. The experimental techniques for the study of the diffusion coefficient mainly include the electrochemical method [5,6]. The electrochemical method can indirectly obtain the diffusion coefficient of rare earth ions through the determination of current, potential and other parameters. The spectroscopy analysis can directly measure the diffusion behavior of rare earth ions in molten salt. However, the complexity of the high-temperature environment, solvation effects and inter-ion interaction often introduce biases into the data and the accuracy of experimental techniques [7,8]. Theoretical calculations and simulations, which mainly include molecular dynamics and first-principles-based computational methods [9,10], are mainly aimed at providing an in-depth understanding of the diffusion mechanism, structure and interactions of rare earth ions in molten salts on the ionic scale. However, due to the non-uniformity, multi-component characteristics and changes at high temperatures of the molten salt system, it is difficult to accurately predict the actual behavior of the ions in the established model, and it still needs to be continuously changed to improve the accuracy and practicality [11]. It is certain that future research needs to continuously optimize the experimental data with the theoretical calculation model in order to further understand the diffusion mechanism of rare earth ions and optimize the related industrial applications.

The market share of NdFeB permanent magnet materials in rare earth applications is about 35–45%. About 50–70% of praseodymium alloy, which is the main master alloy of permanent magnet materials, is produced in fluoride–rare earth oxide system by molten salt electrolysis. The main purpose of this work is to systematically investigate the nature of dissolved Nd* (III) ions transport in the (LiF-CaF_2_)_eut._–Nd_2_O_3_ molten salt system and to provide a guide for the improvement of praseodymium alloys’ electrolytic production efficiency. The diffusion behavior of dissolved Nd* (III) ions in molten salts is closely related to the composition, temperature, solubility, ion mobility, and viscosity of the molten salt. In this study, we adopted a new scheme and strategy to analyze the transport and migration laws of dissolved Nd* (III) ions. We measured the conductivity and ion mobility in a typical (LiF-CaF_2_)_eut._–Nd_2_O_3_ molten salt system and calculated the ion diffusion coefficients of dissolved rare earth Nd* (III) ions with the Nernst–Einstein equation. The interaction laws between ions in the system’s dissolved state were analyzed with the ab initio molecular dynamics (AIMD) method. The results of the study can elucidate the coupled change law between system conductivity, rare earth ion concentration, ion mobility number, temperature, and diffusion coefficient, and provide a more comprehensive theoretical basis for optimizing the transport process of rare earth ions. It is noteworthy that the methodology of the present work can provide a referable idea for the study of the transport properties of electrolytic systems of different rare earth metals and alloys.

## 2. Experimental and Computational Methods

### 2.1. Experimental Methods

Analytically pure LiF, CaF_2_, and Nd_2_O_3_ compounds (Macklin, Shanghai, China) were dried at 473 K for 24 h.

#### 2.1.1. Solubility of Nd_2_O_3_

The solubility of Nd_2_O_3_ in the (LiF-CaF_2_)_eut_ molten salt system was determined using the isothermal saturation method [12]. A homogeneous mixture with a molar ratio of LiF:CaF_2_ = 79:21 and 5 wt. % Nd₂O₃ was heated to a predetermined melting temperature under argon protection. The mixture was stirred and left for 120 min to form the upper saturated layer of the (LiF-CaF_2_)_eut._–Nd_2_O_3_ molten salt. Subsequently, the supernatant of the molten salt was extracted every 20 min using a tungsten capillary tube, then cooled and analyzed by ICP–OES (SPECTRO ARCOS, GERMANY) to determine the Nd content, which was used to calculate the solubility of Nd_2_O_3_.

#### 2.1.2. Measurement of Conductivity and Density of Saturated (LiF-CaF_2_)_eut._-Nd_2_O_3_ Molten Salt

The conductivity of the molten salt system was measured by continuously varying the conductivity cell constant [13]. A tungsten probe connected to a conductivity meter (calibrated with a standard liquid) was immersed in the molten salt contained in a graphite crucible, which was heated to the set melting temperature. After maintaining the temperature for 20 min, the measurement program was initiated to record the data and compute the system’s conductivity using Equation (1). The density of the molten salt system was determined using the Archimedes method [14]. A precision electronic balance was used to measure the mass *M*_1_ (g) of the probe in air and the mass *M*_2_ (g) after immersion in the molten salt, from which the density of the molten salt was calculated using Equation (2).

(1) $$\sigma =\frac{1}{A}(\frac{dL}{dZ})$$

(2) $$\rho =\left(M_{1}-M_{2}\right)/\nu$$

where *σ* is the conductivity (s·cm^−1^); *A* is the internal cross-sectional area of the conductivity cell (cm^2^); *Z* is the total resistance of the circuit (s); *L* is the length of the conductivity cell (cm); *ρ* is the density of the molten salt, g·cm^−3^; *ν* is the volume of the calibrated tungsten probe, cm^3^.

#### 2.1.3. Measurement of Dissolved Nd* (III) Ion Migration Number

According to the definition of an ion migration number, the Nd* (III) ion migration number (*t*_Nd_) is the ratio of the current carried by the Nd* (III) ion to the total current. Therefore, the quantity of Nd* (III) ion migration material (*N*_i_) and the total electric quantity *n*_eq_ passing through the electrode are measured, respectively. The migration number of the Nd* (III) ion can be calculated by *t*_Nd_ = *ZN*_i_ /*n*_eq_. Because the valence of Nd* (III) ion is +3, here, *Z* = 3.

The Hittorf method, interfacial movement method, and electromotive force method are common methods for determining the ion migration number. The Hittorf method is most suitable for estimating the migration number of molten salt ions, which is attributed to the calculated rules of the migration number based on the concentration change near the electrode surface caused by the current passing through the electrolyte.

**Figure 1:**
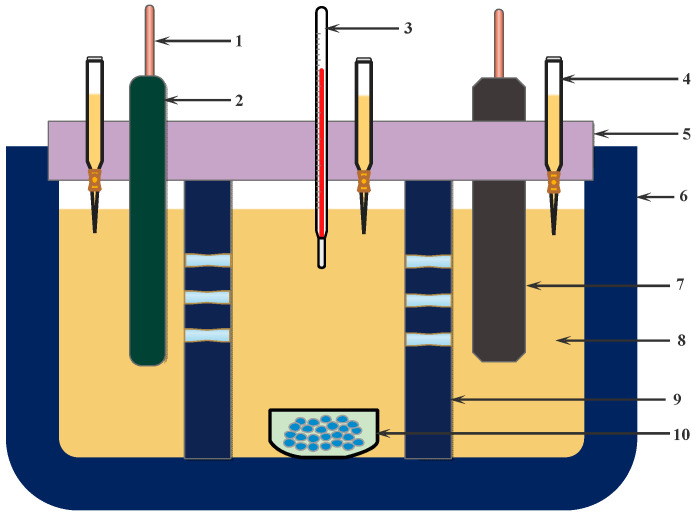
Schematic of the three-compartment cell for migration number measurement. 1. Lead wire; 2. tungsten cathode; 3. thermocouple; 4. capillary suction tube; 5. cover plate; 6. BN crucible; 7. carbon anode; 8. molten salt; 9. partition; 10. tungsten container.

This study employed the Hittorf method to determine the Nd* (III) ion migration number [15]. A three-chamber cell was designed to study the variation in the migration number, comprising anode, cathode, and middle electrode chambers, as shown in Figure 1. The cell was separated by a porous boron nitride plate, with an excess of Nd₂O₃ added to the middle electrode chamber to maintain the (LiF-CaF₂)_eut._–Nd₂O₃ solution in a saturated state. The cell was heated to the calibration temperature under nitrogen protection. After 2 h of thermal equilibrium, a W rod was immersed in the cathode compartment as the cathode, and graphite was used as the anode in the anode compartment. Following constant potential electrolysis, supernatants from the cathode and anode chambers were collected, cooled, and analyzed for Nd content by ICP to determine the concentration change of Nd* (III) ions near the cathode and to quantify the Nd product formation on the tungsten cathode. The amount of Nd* (III) ion migration (*N*_i_) was calculated using Equation (3).

*N*_a_ = *N*_b_ ± *N*_e_ ± *N*_i_ (3)

In this formula, *N*_a_ is the number of Nd* (III) ions after the reaction; *N*_b_ is the number of Nd* (III) ions before the reaction; *N*_e_ is the increase in the number of Nd* (III) ions due to the electrode reaction or the amount of the reduced compound (here, “+” denotes increase, “−” denotes decrease, and 0 represents no n-reaction); *N*_i_ is the number of migrated Nd* (III) ions (“+” denotes “moving in” and “−” designates “moving out”).

### 2.2. Ab Initio Molecular Dynamics Method

The molecules (LiF, CaF_2_, and Nd_2_O_3_) were optimized using the B3LYP [16] approach. A mixed basis set was employed for the computational analysis, wherein Li and F were analyzed using 6–311G* [17,18] basis sets. Yb was examined using the Stuttgart/Dresden pseudo-potential basis set. Packmol (20.14.3) software [19], developed by the University of Campinas in Brazil, was utilized to randomly populate a cubic box of length 145.0 nm with the optimized LiF, NdF_2_, and Nd_2_O_3_ molecules, serving as a preprocessing model. This system comprised 79 LiF and 21 CaF_2_ molecules, alongside one Nd_2_O_3_ molecule, totaling 247 atoms [20]. Periodic boundary conditions were applied, and an NPT ensemble was enforced, employing a Nosé–Hoover thermostat to maintain the target temperature. The pressure and MD time steps were set at 1.013 × 10^5^ Pa (1 atm) and 1 fs, respectively.

Density functional theory (DFT) calculations, conducted with CP2K/Quickstep [21,22] at the Max Planck Institute for Solid-State Research in Stuttgart, Germany, were based on a Gaussian plane-wave approach. Orbital representation was achieved using atom-centered Gaussian-type basis sets, while auxiliary plane-wave basis sets were employed for re-expanding the electron density in reciprocal space. The orbital transformation method facilitated self-consistent field convergence to an accuracy of 10^−6^ Hartree, with all atoms described by the DZVP-MOLOPT-SR-GTH basis set [23]. The valence electrons included the 3d, 4f, and 6s orbital electrons of Nd and those of Li and F, with the remaining electrons accounted for by the Perdew–Burke–Ernzerhof pseudo-potential. The Grimme-D3 dispersion correction [24] was applied, and geometric optimization was conducted using the limited-memory Broyden–Fletcher–Goldfarb–Shanno minimization algorithm [25]. The cut-off and relative cut-off for the electron density, determined using the auxiliary plane-wave basis sets, were set at 600 and 60 Ry, respectively.

## 3. Results and Discussion

### 3.1. Conductivity and Density of Saturated (LiF-CaF_2_)_eut._-Nd_2_O_3_ Molten Salt

**Figure 2:**
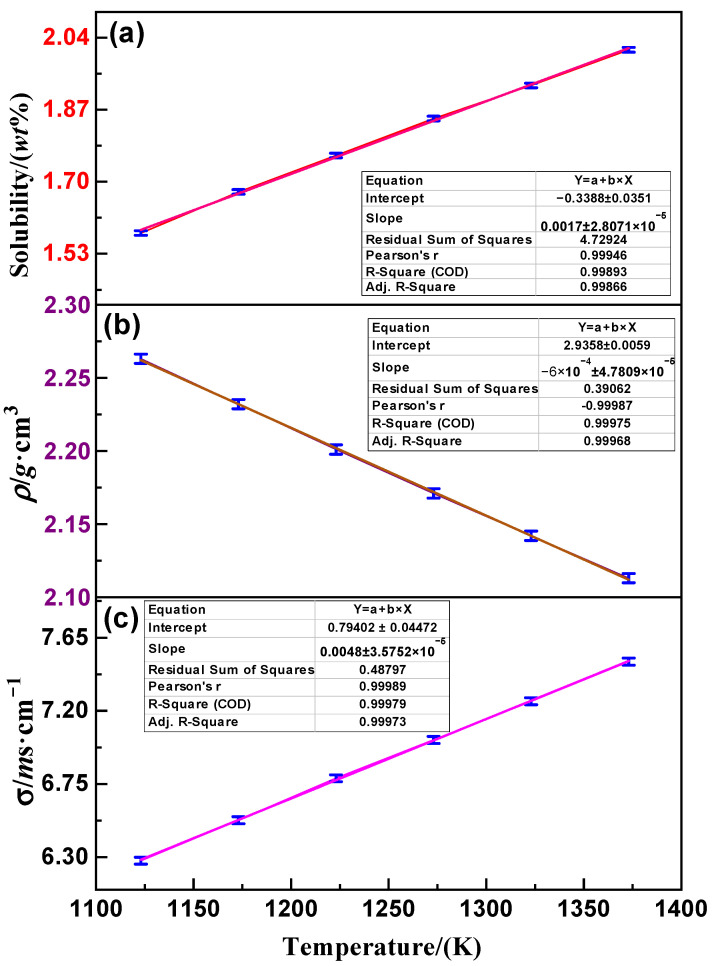
(**a**) Solubility, (**b**) conductivity and (**c**) density variation with temperature for the saturated (LiF-CaF_2_)_eut._–Nd_2_O_3_ system.

The temperature dependence of Nd_2_O_3_ solubility in the (LiF-CaF_2_)_eut._-Nd_2_O_3_ molten salt is depicted in Figure 2a. The solubility of Nd_2_O_3_ in the (LiF-CaF_2_)_eut_ system increases linearly with temperature, ranging from 1173 to 1473 K, within 1.57–2.01 wt. %. This linear relationship is represented by Equation (4). Figure 2b,c display the variations in density (ρ) and conductivity (σ) for the saturated (LiF-CaF_2_)_eut._–Nd_2_O_3_ system over the same temperature range, respectively. The data indicate that the density of the saturated (LiF-CaF_2_)_eut._–Nd_2_O_3_ system linearly decreases with temperature, falling within 2.11–2.26 g·m^3^, consistent with the linear relationship described in Equation (5). Concurrently, conductivity exhibits a linear increase with rising temperature, from 6.28 to 7.50 ms·cm^−1^, aligning with the linear relationship in Equation (6).

(4) $$S_{\mathrm{Nd}2O3}=-0.339+1.71\times 10^{-3}\;T$$

(5) $$\rho_{\mathrm{Nd}2O3}=-2.936-6.00\times 10^{-4}\;T$$

(6) $$\sigma_{\mathrm{Nd}2O3}=-0.794+4.89\times 10^{-3}\;T$$

### 3.2. Migration Number of Solvated Nd* (III) Ions in the Saturated (LiF-CaF_2_)_eut._–Nd_2_O_3_ System

**Figure 3:**
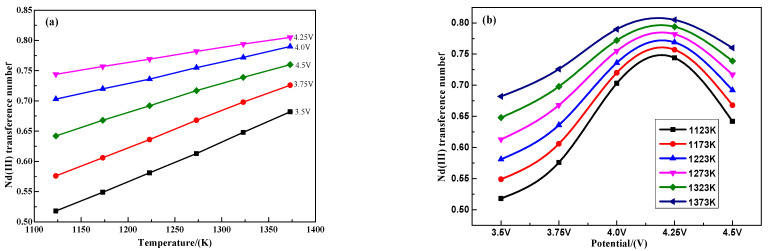
Variation rule of Nd* (III) ion mobility number with (**a**) temperature and (**b**) potential.

Figure 3a illustrates the temperature dependence of the Nd* (III) ion migration number in the saturated (LiF-CaF_2_)_eut._–Nd_2_O_3_ system, with potentials ranging from 3.5 to 4.25 V. It demonstrates that the migration number of Nd* (III) ions linearly increases with temperature between 1123 and 1373 K. Figure 3b reveals that the migration number of Nd* (III) ions in the saturated (LiF-CaF_2_)_eut._–Nd_2_O_3_ system varies nonlinearly with potential within 3.5 to 4.25 V, decreasing after reaching a peak between 4.0 and 4.25 V. This pattern is attributed to the Faraday current peaking as the electrode reduction of Nd* (III) ions on the tungsten cathode surface reaches equilibrium with increasing potential. Concurrently, the system’s free F- and Li+ ions with smaller ionic radii become the non-Faraday current load, reducing the Nd* (III) ion migration number. Moreover, with rising potential, active Nd* (III) ions are intensely polarized at the cathode surface. Other system ions increasingly participate in charge transfer, decreasing the Nd* (III) ion migration number.

**Figure 4:**
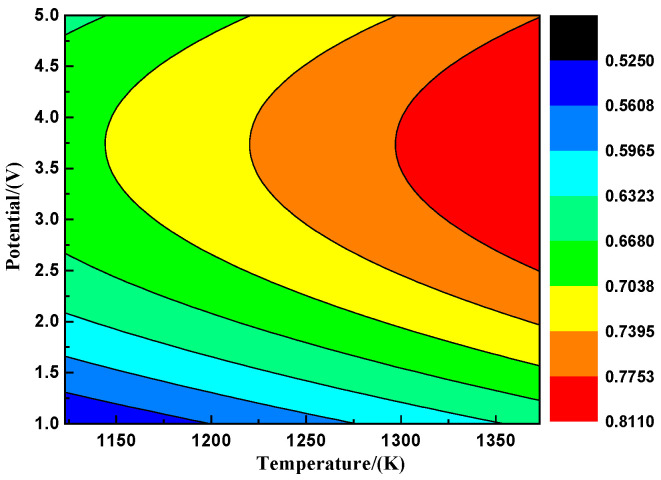
Fitted relationship of Nd* (III) ion mobility number with temperature–potential variation.

Figure 4, derived from the differential fitting of the data in Figure 3, displays the Nd* (III) ion mobility number’s dependence on the temperature–potential relationship in the saturated (LiF-CaF_2_)_eut._–Nd_2_O_3_ system. The Nd* (III) ion mobility number can be maintained at elevated levels at high temperatures (>1300 K) and potentials (>3.0 V). However, high temperatures may destabilize the electrolytic system by increasing molten salt volatilization. The Nd* (III) ion mobility number peaks within the 3.5 to 4.0 V potential range at various temperatures. Using higher potentials (>4.0 V) leads to reduced current efficiency. Overall, the Nd* (III) ion mobility number remains within a high-value range of 0.74 to 0.78 when the system’s temperature and potential are between 1225 to 1300 K and 3.5 to 4.0 V, respectively.

### 3.3. Diffusion Coefficients of Nd* (III) Ions in the Saturated (LiF-CaF_2_)_eut._–Nd_2_O_3_ System

#### 3.3.1. Ab Initio Molecular Dynamics of the Saturated (LiF-CaF_2_)_eut._–Nd_2_O_3_ System

**Figure 5:**
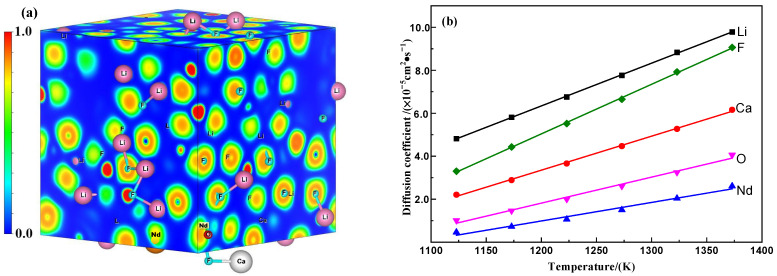
(**a**) Electron localization function diagram and (**b**) variation rule of self-diffusion coefficients of ions with the temperature of the saturated (LiF-CaF_2_)_eut._–Nd_2_O_3_ system.

Figure 5a presents the electron localization function diagram for the saturated (LiF-CaF_2_)_eut._–Nd_2_O_3_ system derived from AIMD calculations. These results reveal the absence of distinct electron-density domains, suggesting that the metallic (Li, Ca, Nd) and nonmetallic (O, F) elements predominantly exist in ionic forms within the system, denoted as M*, where M = Li/Ca/O/Nd/F. The primary interaction among these ions is electrostatic.

Utilizing AIMD computations and monitoring the ionic trajectories over time, one can determine the atoms’ root-mean-square displacements (MSDs), which reflect the positional changes relative to a reference point. These MSDs, calculated using Equation (7), facilitate the computation of the self-diffusion coefficients of ions in the saturated (LiF-CaF_2_)_eut._–Nd_2_O_3_ at various temperatures, based on Einstein’s Equation (8). As depicted in Figure 5b, the self-diffusion coefficients of ions M* in the system exhibit a linear temperature-dependent increase. The order of inter-ion diffusion coefficients is *D* (Li) > *D* (F) > *D* (Ca) > *D* (O)> *D* (Nd), with a decrease in metal ion self-diffusion coefficients as the atomic number increases (*D*(Li) >*D*(Ca) > *D*(Nd)). Notably, F (I) ions demonstrate higher diffusion coefficients than O (II) ions (*D* (F) > *D* (O)). Transforming the Arrhenius Equation (9) of the diffusion coefficient into a logarithmic relationship (Equation (10)) and performing a linear fit of ln*D*-(1/*T*) yields the diffusion activation energies and pre-exponential factors for various ions, detailed in Table 1. These data suggest a descending order of M* ion diffusion activation energies—*E* (Nd) > *E* (O) > *E* (Ca) > *E* (F) > *E* (Li), with Nd exhibiting the highest atomic mobility barriers and Li the lowest.

(7) $$MSD=\langle {\left|x\left(t\right)-x_{0}\right|}^{2}\rangle =\frac{1}{N}\sum_{i=1}^{N}\langle {\left|x^{\left(i\right)}\left(t\right)-x^{\left(i\right)}\left(0\right)\right|}^{2}\rangle$$

(8) $$D=\underset{t\to \infty }{\lim}\left[\frac{1}{2dt}\langle {\left|x\left(t\right)-x_{0}\right|}^{2}\rangle \right]$$

(9) $$D=D_{0}exp\left(-\frac{E}{RT}\right)$$

(10) $$lnD={lnD}_{0}-\frac{E}{RT}$$

where *d* is the MSD dimension, which is three-dimensional in this paper; *t* is the time; $\langle {\left[\vec{r}\left(t\right)\right]}^{2}\rangle$ is the mean-square displacement; *D* is the diffusion coefficient of the ions, cm^2^·s^−1^; *E* is the activation energy of ion diffusion, J·mol^−1^; *R* is the ideal gas constant, 8.314 J·mol^−1^·K^1^.

**Figure 6:**
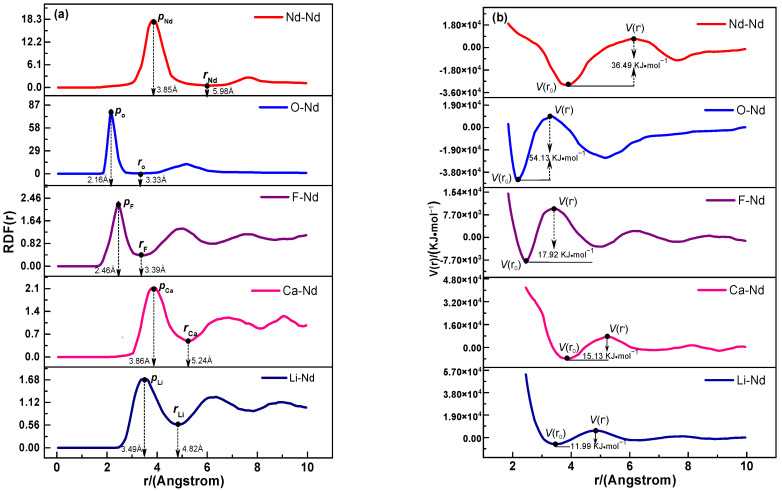
(**a**) Radial distribution function and (**b**) migration energy barriers of M*-Nd* (III) in the –saturated (LiF-CaF_2_)_eut._-Nd_2_O_3_ system.

To further examine the interaction strength and migration energy barriers of Nd* (III) ions with M* ions in the saturated (LiF-CaF_2_)_eut._–Nd_2_O_3_ system across the temperature range 1123–1373 K, the high-temperature kinematic trajectory data from AIMD were scrutinized. This analysis yielded the radial distribution function (RDF, *g* (*r*_M_*)) between M* ions and Nd* (III) ions. Figure 6a depicts the RDF curve (mean value) for M* and Nd* (III) ions within the specified temperature range. The migration energy barriers between M* ions and Nd* (III) ions were determined using Equation (11) and are illustrated in Figure 6b. Table 2 presents the average first coordination radius (*R*_M*_), truncation radius (*r*_M*_), and migration energy barriers *V* (M*) between Nd* (III) ions and the corresponding M* ions in the system.

(11) $$V\left(r\right)=-RTlng(r)$$

where *r* is the ideal gas constant 8.314 J·mol^−1^·K^1^; *T* is the absolute temperature, K; *g* (*r*) is the value of the radial distribution function between ions.

The average first coordination radius (*R*_M*_) and truncation radius (*r*_M*_) for each central ion M* with Nd* (III) ions, as illustrated in Figure 6a and Table 2, reveal that the anion F* (I) possesses a smaller first coordination and truncation radius compared to the O* (II)-Nd* (III) ions (*R*_O*_ = 2.16 Å, *r*_O*_ = 3.33 Å). This suggests a more compact and regular first coordination layer for O* (II)-Nd* (III) ions, attributable to the stronger charge effect of O* (II) on Nd* (III) compared to F* (I). Consequently, O* (II) exerts a more significant influence on the diffusive migration of Nd* (III) ions. The first coordination radius closely matches the truncation radius between the system cations [Nd* (III)/Ca* (II)/Li* (I)] and Nd* (III). Despite stronger charge interactions between Nd* (III)-Nd* (III) and Nd* (III)-Ca* (II), the first coordination layer of Nd* (III)-Li* (I) is tighter due to Li* (I)’s smaller radius. The first coordination and truncation radii between cations [Nd* (III)/Ca* (II)/Li* (I)] and Nd* (III) are larger than those between anions [O* (II)/F* (I)] and Nd* (III), resulting in a looser first coordination layer.

Analysis of the data from Figure 6b and Table 2 indicates that the average migration energy barrier V (M*) for ions in the system follows the order V (O*) > V (Nd*) > V (F*) > V (Ca*) > V (Li*). This suggests that O* (II) ions have the strongest influence on Nd* (III) ions during diffusive migration, while Li* (I) exerts the weakest. The interaction between Nd* (III) ions is stronger than that between F* (I) and Nd* (III) due to a higher nuclear charge and stronger electrostatic interactions. Given Nd* (III)’s larger ionic radius, its diffusive migration between ligand layers represents a complete homoionic substitution with limited inter-ionic free volume. In contrast, F* (I) has a smaller ionic radius, resulting in a larger interionic free volume for Nd* (III)-F* (I) during diffusion migration.

#### 3.3.2. Calculation and Analysis of Diffusion Coefficients of Nd* (III) Ions

Using the conductivity and mobility data of the saturated (LiF-CaF_2_)_eut._–Nd_2_O_3_ system and the calculated self-diffusion coefficient of Nd* (III) ions, we can ascertain the diffusion characteristics and laws of active Nd* (III) ions in the system. This enables the electrolysis process to optimize the “potential–temperature” matching region.

The Nd* (III) ion diffusion coefficient (*D*_Nd_) expression (13) of the saturated (LiF-CaF_2_)_eut._–Nd_2_O_3_ system is derived from the Nernst–Einstein Equation (12). Utilizing the data of conductivity, solubility and density from Figure 1 and the migration number of Nd* (III) ions from Figure 3, the Nd* (III) ion concentration at these temperatures can be calculated. Subsequently, the Nd* (III) ion diffusion coefficients are computed from Equation (13) under different temperatures and potential conditions.

**Figure 7:**
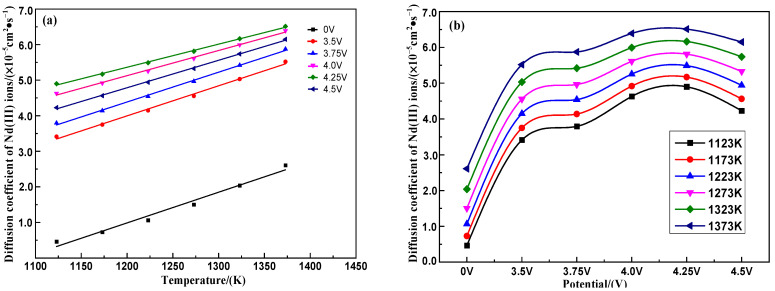
Changes of Nd* (III) ion diffusion coefficients with (**a**) temperature and (**b**) potential in the saturated (LiF-CaF_2_)_eut._–Nd_2_O_3_ system.

Incorporating the Nd* (III) ion self-diffusion coefficient of the saturated (LiF-CaF_2_)_eut._–Nd_2_O_3_ system calculated in Figure 5b at 0 V potential, the relationship between the Nd* (III) ion diffusion coefficient, temperature and potential is illustrated in Figure 7. It reveals a linear increase in the Nd* (III) ion diffusion coefficient with temperature (1123–1373 K) under fixed potential conditions (0–4.5 V). The diffusion coefficient of Nd* (III) ions exhibits nonlinear variation with increased potential (0–4.5 V) within this temperature range, peaking and then declining in the range 4.0–4.5 V. In the 0–3.50 V range, Nd* (III) ions were subjected to a stronger electric field and the diffusion coefficient increased as the potential increased. When the voltage was 3.50–4.25 V, the electric reduction of Nd* (III) ions on the tungsten cathode surface reached equilibrium, the concentration gradient of Nd* (III) ions reached their extreme value, and the diffusion coefficient also reached its extreme value. When the potential exceeded 4.25 V, the concentration gradient of Nd* (III) ions gradually decreased, and the free Li^+^/Ca^2+^ ions became carriers of a non-Faraday current, which led to a decrease in the diffusion coefficient of Nd* (III) ions.

The diffusion activation energies of Nd* (III) ions at various potentials, determined from the logarithmic transformation of the temperature–diffusion coefficient linear fitting equation in Figure 7a, are presented in Table 3. These data indicate that the Nd* (III) ion diffusion activation energy at potentials of 4.0 V or 4.5 V is higher than at 4.25 V, suggesting the lowest diffusion barrier for Nd* (III) ions occurs in the range 4.0–4.5 V.

**Figure 8:**
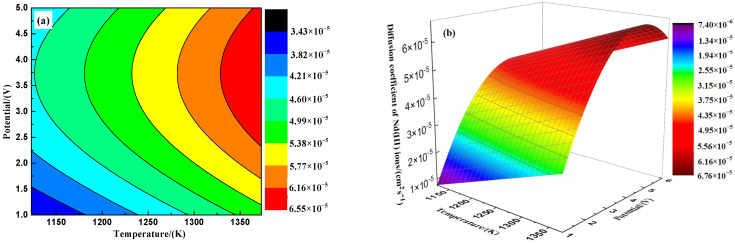
(**a**) Two-dimensional fitting diagram and (**b**) three-dimensional surface fitting diagram of Nd* (III) ion diffusion coefficient with temperature–potential variation.

The further differential fitting of the Nd* (III) ion diffusion coefficients from Figure 7 enables the depiction of the “temperature–potential” coupled diffusion coefficient trend, as shown in Figure 8. The two-dimensional fit in Figure 8a indicates that the highest diffusion rate for Nd* (III) ions is maintained at a control potential above approximately 2.25 V and a temperature above approximately 1325 K. The three-dimensional surface fit in Figure 8b demonstrates a rapid and sensitive potential response of the Nd* (III) ion diffusion coefficient.

(12) $$\sigma =\frac{C_{i\;}D_{i\;}Z_{i}^{2}F^{2}}{t_{i}RT}\;$$

(13) $$D_{i}=(9.92\times 10^{-11})\cdot \frac{\sigma t_{i}T}{C_{i\;}}$$

where *σ* is the conductivity; *C*_i_ is the concentration of ions, mol·L^−1^; *D*_i_ is the diffusion coefficient of ions, cm^2^·s^−1^; *Z*_i_ is the charge number of ions, which is taken as 3 in this paper; *F* is the Faraday’s constant, 96,485 C·mol^−1^; and *t*_i_ is the migration number of ions.

## 4. Conclusions

In the saturated (LiF-CaF_2_)_eut._–Nd_2_O_3_ system, the solubility and conductivity of Nd_2_O_3_ exhibit linear increases within the 1173–1473 K temperature range, whereas density decreases linearly. The mobility of solvated Nd* (III) ions in the saturated (LiF-CaF_2_)_eut._–Nd_2_O_3_ system linearly increases over the temperature range of 1173–1473 K. However, it demonstrates a nonlinear variation within the potential range of 3.5–4.5 V under specific temperature conditions, decreasing after peaking in the 4.0–4.25 V interval. The diffusion coefficient of solvated Nd* (III) ions at a specific potential (3.5–4.5 V) linearly rises with temperature in the range 1123–1373 K. At fixed temperatures (1123–1373 K), the diffusion coefficients vary nonlinearly with potential (3.5–4.5 V) and decline after reaching a maximum in the 4.0–4.5 V range, indicating a rapid potential response in the diffusion coefficients of Nd* (III) ions.

The radial distribution function analysis using ab initio molecular dynamics indicates that the diffusion activation energies of solvated M* ions in the saturated (LiF-CaF_2_)_eut._–Nd_2_O_3_ system are ranked as follows: E (Nd) > E (O) >E (Ca) > E (F) > E (Li). The solvated Li* (I) ion exhibits the lowest migration energy barrier, whereas the solvated Nd* (III) ion has the highest. Solvated O* (II) ions exert the strongest pull on Nd* (III) ions. Furthermore, Nd* (III) ions maintain a high diffusion rate at a control potential of approximately 2.25 V and a temperature of approximately 1325 K.

## Figures and Tables

**Table 1:** Linear fitting relationship of diffusion coefficients (*D*), temperature (*T*) and lnD ~ 1/T for M* (M = Li,Ca,Nd,O,F) ions and diffusion kinetic parameters (prefactor and diffusion activation energy (DEA)).

Ionic Species	Diffusion Coefficient Fits	ln*D* ~ 1/*T* Fit	Prefactor (J·mol^−1^)	DEA(J·mol^−1^)
Li(I)	$D_{\mathrm{Li}}=-$1.75 × 10^−4^ + 1.99 × 10^−7^*T*	Y=−6.05 − 4357.29X	2.36 × 10^−3^	36,226.51
Ca(II)	$D_{\mathrm{Ca}}=-$1.56 × 10^−4^ + 1.58×10^−7^*T*	Y=−5.08 − 6310.64X	6.22 × 10^−3^	52,466.66
Nd(III)	*D*_Nd_ = 9.37 × 10^−5^ + 8.63 × 10^−8^*T*	Y=−2.74 − 10,677.55X	6.46 × 10^−2^	88,773.15
O(II)	$D_{O}=-$1.27 × 10^−4^ + 1.21 × 10^−7^*T*	Y=−3.90 − 8497.52X	2.02 × 10^−2^	70,648.38
F(I)	$D_{F}=-$2.27 × 10^−4^ + 2.31 × 10^−7^*T*	Y=−4.77 − 6186.31X	8.48 × 10^−3^	51,432.9

**Table 2:** Average coordination radius, Cut-off radius and average migration energy barrier (V (r)) of M* ((M = Li,Ca,Nd,O,F)-Nd*in the (LiF-CaF_2_)_eut._–Nd_2_O_3_ system.

M*-Nd*	Average Coordination Radius/Å	Cut-Off Radius/Å	V(r)/KJ·mol^−1^
Li-Nd	3.49	4.82	11.99
Ca-Nd	3.86	5.24	15.13
F-Nd	2.46	3.39	17.92
O-Nd	2.16	3.33	54.13
Nd-Nd	3.85	5.98	36.49

**Table 3:** Linear fitting relationship of diffusion coefficients (*D*), temperature (*T*) and lnD~1/T for Nd* (III) ions and diffusion kinetic parameters (Prefactor and diffusion activation energy (DEA)) with temperature at different potentials.

Potential(V)	Diffusion Coefficient Fits	lnD ~ 1/T Fit	Prefactor (J·mol^−1^)	DEA (J·mol^−1^)
0	*D* = −9.37×10^−5^ + 8.63×10^−8^*T*	Y = −2.74 − 10,677.56X	6.46×10^−2^	88,773.23
3.5	*D* = −6.14×10^−5^ + 8.45×10^−8^*T*	Y = −7.65 − 2974.74X	4.76×10^−4^	24,731.99
3.75	*D* = −5.67×10^−5^ + 8.38×10^−8^*T*	Y = −7.77 − 2716.0X	4.22×10^−4^	22,580.82
4.0	*D* = −4.49×10^−5^ + 7.73×10^−8^*T*	Y = −8.21 − 1998.09X	2.72×10^−4^	16,612.12
4.25	*D* = −3.37×10^−5^ + 7.08×10^−8^*T*	Y = −8.36 − 1766.48X	2.34×10^−4^	14,686.51
4.5	*D* = −2.44×10^−5^ + 6.50×10^−8^*T*	Y = −8.01 − 2322.01X	3.32×10^−4^	19,305.19

## Data Availability

The original contributions presented in the study are included in the article.
